# Evaluation of virtual training delivery for health information systems implementation in Canada: A qualitative study

**DOI:** 10.1177/18333583241289151

**Published:** 2024-11-20

**Authors:** Inaara Karsan, Hafsa Hasan, Tharshini Jeyakumar, Sharon Ambata-Villaneuva, Katharine Fur, Ivanka Hanley, Sarah McClure, Maram Omar, Tamee Sheriff, David Wiljer

**Affiliations:** 1 University Health Network, Canada; 2 University of Toronto, Canada

**Keywords:** health information system, education, training, virtual, inclusivity, sustainment, healthcare providers, patient care

## Abstract

**Introduction:**

As health information systems (HIS) become a critical part of patient care, it is crucial to build an effective education strategy that facilitates the adoption and sustained use of these systems. The COVID-19 pandemic (2019–2023) has contributed to the rapid shift in virtual education and training for healthcare staff.

**Objective:**

We sought to evaluate the efficacy and long-term sustainability of virtual training for using a HIS by examining opportunities and challenges.

**Method:**

An exploratory, multimethods study was conducted with staff who had taken part in a virtual HIS training program as part of the clinical transformation journey at a large academic health science center in Canada. The study was guided by the Accelerating the Learning Cycle framework. Data were collected through pre- and post-training surveys, as well as semi-structured interviews. An iterative, inductive, constant comparative analysis approach, outlined by Braun and Clarke, was taken to thematically analyse the data.

**Results:**

Of the 33 participants in this study, 13 were educational champions, and 20 were end-users. The pre- and post-training surveys yielded a total of 1479 responses in both groups. Three prominent themes emerged from this study: (1) fostering dynamic facilitation techniques to cultivate an inclusive culture and adapt to diverse learning needs; (2) integrating practical learning activities that contribute to knowledge retention; and (3) ensuring training resources are accessible and consistent for an optimal training experience.

**Conclusion:**

As HIS continue to be part of the transformation of the healthcare ecosystem, education is vital in preparing healthcare providers to perform their clinical tasks and effectively use these technologies. Findings from this study can be used to inform the development of virtual training that is inclusive and addresses the needs of care providers.

## Introduction

Health information systems (HIS) have attracted the attention of many healthcare organisations to facilitate clinical transformation necessary for healthcare innovations and improving patient outcomes. The formation of Canada Health Infoway in 2001 led to the acceleration of digital health solutions including HIS in Canada. Similarly, the United States (US) government introduced the Meaningful Use program as part of the *Health Information Technology for Economic and Clinical Health Act* in 2009 to encourage organisations to adopt the use of HIS and improve the quality of patient care (Henricks, 2011). The implementation of HIS better positions healthcare organisations to improve all aspects of patient care, including safety, patient-centeredness, communication and equity (Bredfeldt et al., 2013; Bygholm, 2018; Edwards et al., 2012). Additionally, it mobilises efforts to create operational efficiencies for physicians and work towards interoperability between hospitals.

HIS is an umbrella term used to describe an integrated information system that enables the collection, processing, reporting, and use of information to guide decision-making and research activities in health care (Almunawar and Anshari, 2012; Jakovljević, 2008). Hospital information systems are a type of HIS, specifically targeted for use in hospital settings and healthcare providers (Epizitone et al., 2023; Winter et al., 2023). Electronic medical records are a central component within a HIS, consisting of patients’ medical records in an electronic format (Almunawar and Anshari, 2012). On the other hand, electronic health records are defined as the collection of an individual’s health information from various healthcare institutions (Winter et al., 2023). As the focus of the study is on education of any HIS for healthcare professionals, the term “HIS” will be used throughout the article.

Implementing a HIS is recognised as a large-scale process as it not only relies on the technical processes but also involves participation across a whole organisation (Bygholm, 2018). Studies evaluating the effectiveness of HIS educational approaches have emphasised the importance of investing in understanding the learners in the clinical context, maximising problem-based learning (PBL) experiences to transfer learning to care, and engaging in continuous evaluation to meet the emerging demands of the clinical environment (Bredfeldt et al., 2013; Edwards et al., 2012; McCain, 2008; Pantaleoni et al., 2015; Vuk et al., 2015). Specifically, adequate training and support, sufficient time for training, and digital literacy are highlighted as human and social critical factors for implementing a HIS across an organisation (Bygholm, 2018). However, a challenge in developing HIS education identified in studies was ensuring that the content was relevant and applicable to all learners, as individual digital skills vary among staff. Therefore, strategic planning to execute training and education is necessary to ensure staff adoption and sustained use of a HIS. Many studies have highlighted the importance of training to support a HIS implementation and have rigorously outlined how to adapt training to meet learners’ needs (Bygholm, 2018; Pantaleoni et al., 2015; Vuk et al., 2015). In the literature, methods of delivery for HIS training have varied and included instructor-led, web-based, simulation and blended learning approaches to educate staff (Jeyakumar et al., 2021). Although there are various methods to conduct HIS training, a scoping review outlined an education model consisting of five fundamental elements that contribute to an effective education strategy for HIS implementation (Jeyakumar et al., 2021). Jeyakumar et al. (2021) found effective education approaches consisted of (1) engaging learners to understand their clinical context, (2) engaging in PBL, (3) maximising the relevance of the training content to the clinical care, (4) involving “super users” early to help foster value of HIS within teams, and (5) integrating continuous evaluations to identify emerging needs of the clinical environment.

However, the COVID-19 pandemic highlighted challenges in the current training strategies and introduced a variety of unprecedented learning requirements (Chew and Sim, 2021; Hall et al., 2020). The pandemic disrupted healthcare systems not only affecting the delivery of care but also the delivery of training and education. There has been a shift towards adopting virtual training as the primary modality to support training initiatives within healthcare organisations (Hall et al., 2020). Virtual training offers advantages such as reaching a large learner population while also upholding the distancing regulations and translating core skills and competencies to learners. Despite recommendations for healthcare organisations to engage in virtual training during the pandemic, formal HIS virtual training programs have not been widely established or studied (Consorti et al., 2021; Hall et al., 2020). Research has emphasised the importance of a culturally responsive and inclusive learning environment to facilitate training (Pantaleoni et al., 2015), which includes training occurring in locations close to the staff’s clinical practice setting, ensuring flexible scheduling and assessing digital literacy. However, strategies on how to translate these principles into a virtual environment remain under-researched (Mohan et al., 2021). A knowledge gap exists regarding the optimal design, development and use of training tools to support learners in this new context of training delivery for a HIS implementation (O’Doherty et al., 2018).

To embark on the clinical transformation journey of implementing a HIS during a pandemic, our organisation has leveraged virtual training for all HIS education initiatives. The education strategy consisted of a phased approach for all staff across four hospital sites. While the organisation is embracing digital technology to support clinical transformation, it is important to assess the effectiveness of virtual training among staff learners. The objective of this study was to evaluate the dimensions of virtual training effectiveness for a HIS implementation by examining the opportunities and challenges for facilitating training in a virtual environment and providing recommendations to improve virtual education activities.

## Method

### Research design and context of the study

This study describes a large-scale initiative at a large academic health science centre (the Centre), which transitioned from a hybrid approach involving electronic patient records and paper-based documentation and orders to a single integrated system, enabling improved communication among care teams and patient-centred care. The Centre is located in Toronto, Ontario, serving four hospitals and one academic centre, with approximately a total of 17,000 staff.

The study was conducted from January 2022 to August 2022. The research design incorporated an exploratory, multimethods qualitative approach, theoretically informed by the *Accelerating the HIS Learning Cycle* framework to evaluate the effectiveness and long-term sustainment of virtual HIS training (Jeyakumar et al., 2021). The HIS learning cycle framework served as a guide for the design and development of HIS education during and post-implementation (Jeyakumar et al., 2021). This framework is cyclical and consists of five key elements: (1) assessment of individual, team and organisational capabilities; (2) practice and PBL; (3) integrate learning into practice; (4) enhance practice improvement and performance; and (5) evaluation and feedback (Jeyakumar et al., 2021). The first element emphasises the need to assess the current capability of providers and clinical teams, recognising that HIS knowledge and adoption levels may vary (Jeyakumar et al., 2021). Education programs should be designed using a PBL approach that fosters independent learning, critical reflection, and the application of problem-solving skills to key concepts learned (Jeyakumar et al., 2021). Additionally, the framework underscores the importance of integrating learning into practice by providing opportunities for hands-on practice and real-life case scenarios (Jeyakumar et al., 2021). Thus, the case examples emulate the clinical environment, encouraging learners to understand clinical workflows and navigate unfamiliar contexts (Jeyakumar et al., 2021). The ‘enhance practice improvement and performance’ element stresses the need for clinical champions in shifting the mindset towards technology (Jeyakumar et al., 2021). Finally, it is crucial for HIS education programme to be continuously evaluated and adapted to address ongoing learning needs (Jeyakumar et al., 2021; McLoughlin, 2001). As part of the evaluation element of the HIS learning cycle, the study findings were situated within the educational outcomes identified in the Kirkpatrick–Barr model (Shen et al., 2017). This evaluation model assesses educational outcomes based on four hierarchical criteria: (1) learners’ reaction; (2) modification of attitudes and acquisition of knowledge; (3) change in behaviour and (4) changes in organisational practice (Shen et al., 2017).

As this study was approved as a quality improvement project, it was granted an exemption by the institution’s Research Ethics Board (QI ID: 21-0325).

### Educational intervention

**Figure 1.**
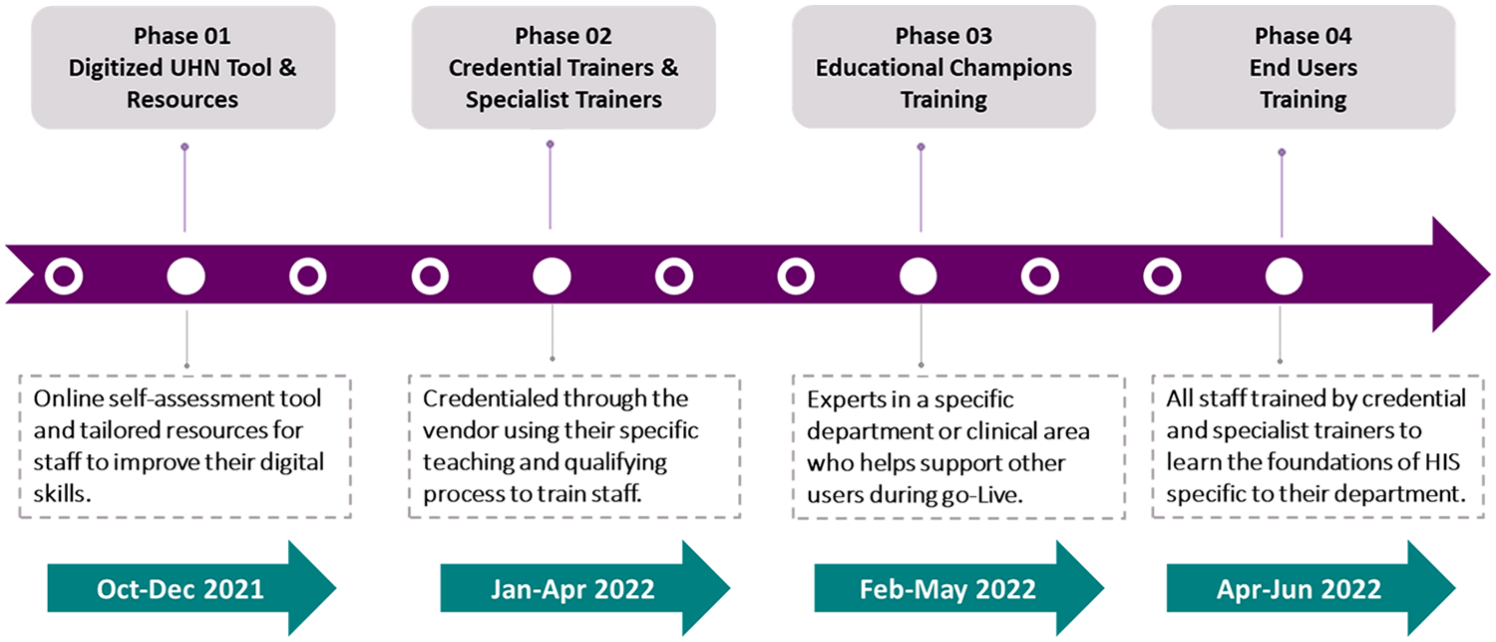
HIS training process.

This educational intervention included a 4-phase education approach, incorporating adult learning theory and multimodal education tools (Figure 1). Principal educators were responsible for the overall curriculum design and development. They supported their application group of trainers to be fully equipped to conduct the HIS training for educational champions and end-users. Principal educators were certified by the HIS vendor in specialty areas related to HIS during which they were tasked with designing a curriculum. They tailored and customised content and made scripts for lesson plans rolled out to the credential trainers after their onboarding.

Phase 1 focused on developing an online self-assessment tool that enabled staff to assess their digital skills and access tailored resources. This also ensured staff were prepared and well-equipped to take virtual training. Phase 2 was designed to orient training specialists and peer educators to the system. They were assigned a specific system application to train alongside a peer educator. Peer educators were trained by the training specialists playing a dual role as an educational champion, specialising in their role, and working closely with the principal educators. Some peer educators also had learning experiences through previous HIS implementation at other hospitals. They worked closely with education specialists to help build templates and assist in providing feedback to different workflows and specialties. Phase 3 was designed to orient educational champions to the system. An educational champion is an expert in a specific department, clinical area or subject matter content area, who helps support other users in that area. Educational champions are one of the organisation’s most valuable assets during end-user training and go-live because they combine knowledge of the new system with experience using the organisation’s legacy systems and processes. In phase 4, end users were trained to use the system. All staff were required to complete training in order to receive access to the system.

Training sessions were provided virtually using a videoconferencing platform, Microsoft Teams, and were approximately 4–6 hours in length, with mini didactic lectures and practice opportunities. During each session, two facilitators were present to communicate the content and to provide technical support. Learners were provided with resources consisting of a class companion, an exercise booklet and a class information sheet. E-learning was self-directed and optional, categorised based on specific roles. Staff were required to register and attend the necessary virtual sessions. A sandbox environment^1^ was available to staff to practice workflows and role-based scenarios using the HIS interface. The sandbox environment provided a safe space to explore the interface without the fear of error, enhanced learning opportunities, and hands-on experience and allowed for knowledge reinforcement. All staff had to pass an assessment after training in order to be granted permission and access to use the system.

### Study setting and selection of participants

A maximum variation purposive sampling method was used to gain a wide variety of perspectives that should be considered in the implementation of a HIS (Patton, 1990). Participants included all staff across all hospital sites who took part in virtual instructor-led training sessions and provided informed consent. The hospital sites specialised in acute care, outpatient, cancer care and rehabilitation services. Participants were recruited through email invitations sent by the research analyst. Electronic gift cards were sent as an appreciation upon the completion of the interviews.

## Data collection

### Pre- and post-training surveys

The data collection process consisted of a pre- and post-training survey. The pre-training survey assessed staff readiness for virtual training and the level of confidence with the transition prior to go-live. All participants who attended the training were sent a web-based post-training survey to complete after the training. The post-training survey consisted of two sections highlighting (1) thoughts about the training session, and (2) the usability of the educational activities offered online. REDCap, an online survey tool, was used to disseminate the surveys.

### Semi-structured interviews

Virtual one-on-one interviews were conducted and recorded through the Microsoft Teams platform by two research analysts and were, on average, 30–40 minutes in duration. A semi-structured interview guide was used as it enabled an in-depth understanding of the training programme and allowed space for expressing individual experiences. The interview guide was informed by the HIS learning cycle framework and consisted of 12 open-ended questions. All participants provided verbal informed consent. The recordings were professionally transcribed verbatim.

### Data analysis

Interviews were analysed by a data analysis software tool (NVivo, QSR International). The research team deductively interpreted the findings using the first three levels outlined in the Kirkpatrick–Barr model (Barr et al., 2000) to determine effective strategies to engage learners. The fourth level of organisational practice change was not assessed in the duration of the current study. An iterative, inductive, and constant comparative process was used in addition to collaboratively create an initial coding structure and shape further data collection by examining the recurring and emerging themes from both the interviews and surveys. The HIS Learning Cycle framework was also used to reflect on the evaluation results and the effectiveness of HIS training, as it was a key pillar of the education design. Three research analysts trained in qualitative research analysed the data using a systematic process as outlined by Braun and Clarke (2006). A senior investigator was consulted when consensus could not be reached. The initial codes and themes were discussed with the research team and iteratively refined throughout the process. Data collection and analysis continued until saturation was reached (Saunders et al., 2018).

## Results

The survey yielded 211 responses out of a total of 2193 educational champions, and 1268 responses out of a total of 13,651 end users. Educational champions were characterised as early adopters of HIS training in their clinical area, whereas end users consisted of staff including clinicians, administrators and support staff from varied clinical areas. Survey responses identified that educational champions (at 74%) and end users (at 60%) were highly satisfied with the overall experience of the training respectively. Among educational champions, 68% reported that they were prepared for the HIS implementation after training, and 45% were confident or highly confident in using the system. End users reported similar findings, with 70% of participants reported feeling prepared to use the system and 40% reported feeling confident or highly confident in using the HIS.

A total of 33 participants (13 educational champions and 20 end users) agreed to participate in semi-structured interviews. Of the 33 participant interviewees, various roles were covered including technicians, clinical coordinators, transplant coordinators, nurses, financial analysts, allied health professionals, administrative assistants, mental health professionals, pharmacists, privacy analysts and health information specialists. Staff were receptive to the virtual delivery of HIS training. Three major themes emerged from the interviews and survey responses to help learners progress through Kirkpatrick–Barr levels: (1) fostering dynamic facilitation techniques to cultivate an inclusive culture and adapt to diverse learning needs; (2) integrating practical learning activities that contribute to knowledge retention; and (3) ensuring training resources are accessible and consistent for an optimal training experience (Table 1).

**Table 1.** Outline of themes and subthemes.

Key themes and subthemes
Theme 1: Foster dynamic facilitation techniques to cultivate an inclusive culture and adapt to diverse learning needs • Develop a learner-centred experience by optimising the virtual environment • Foster a receptive and engaging learning environment • Maintain a reactive and adaptive training approach based on learners’ needs to reduce cognitive overload • Promote learner engagement by adopting an energetic personality and utilizing balanced facilitation techniques • Adopt a peer-to-peer training approach
Theme 2: Integrate practical learning activities that contribute to knowledge retention • Sandbox enhanced learning opportunities • Ensure readiness of virtual training environment • Promote hands-on experiences to enhance learning opportunities • Develop reinforcement learning approaches
Theme 3: Ensure training and resources are accessible for optimal training experience • Deliver consistent training format and communication • Provide accessible technical support • Provide accessible technology resources and learning space

### Theme 1: Fostering dynamic facilitation techniques to cultivate an inclusive culture and adapt to diverse learning needs

#### Develop a learner-centred experience through optimising the virtual environment

Participants felt that a comfortable and inclusive learning space was developed through integrating core activities such as establishing ground rules, facilitating introductions and participating in icebreakers. These activities provided an opportunity to feel comfortable in the virtual classroom and more willing to participate during the training session. Some participants noted that since no physical room exists in a virtual environment, it is critical learners feel a sense of togetherness and community in the virtual spaces:

Sure, actually, it was a really great space for learning. Both educators, you know, introduce themselves fully and what to expect out of the session. They then asked us to introduce ourselves. So even though you know, it was a team of dietitians, we all work in different areas. So, it was kind of nice to have that opening piece, I guess. The intros about each other and then, yeah, it was a fairly easy process. People were respectful. (End User 13)

Interestingly, those who participated in more than one training session acknowledged that smaller class sizes improved learner engagement because it was easier to ask questions and have in-depth discussions:

Um, I think the small groups are great, I think that needs to be maintained. I want to say there was maybe six in my group, you know, but just allows for just the right amount of questions with not, you know, slowing down the teach process, but also enough people where you can kind of collaborate and, you know, discuss something that somebody has a question about, somebody else will chime in and say, well, actually, we have to also do referrals at our area. So, this is what I’ve heard. And you could kind of help educate each other on different things that we’ve learned, or we were told. And so, it became a very collaborative session. So, I think the smaller groups are good. (End User 13)

Participants reflected that the virtual platform was effective for connectivity and collaboration; however, limitations were associated with visualising and emphasising the features of the interface on their screen. Facilitators used the platform features to increase accessibility, which improved the participants’ learning experience. Specifically, a few participants noted that the facilitators increased the cursor size, used annotations on the slides, or used digital tools embedded in the platform to assist the direction of the learner’s attention. HIS training that addressed learners’ needs by fostering an inclusive learning environment through activities, smaller group sizes, and ensuring the content is visually accessible, allowed for a constructive training session.

#### Maintain a reactive and adaptive training approach based on learners’ needs to reduce cognitive overload

Participants felt that training was conducted at a fast pace, which led to a lack of knowledge retention and cognitive overload. Specifically, participants reflected that some of the courses contained a lot of information in a short amount of time which led to facilitators adopting a faster pace to ensure that they cover all the necessary content. Although some facilitators did use verbal cues from the learners to adjust their content and pace to be applicable to the group, some participants commented that the virtual learning environment hinders an adaptive approach due to the absence of visual cues. Most participants that took part in training reported that they were not on camera. In this way, facilitators cannot visualise the body language and reactions of participants to alter their approach or modify support:

We were told, oh, but we don’t have time for that right now. You know, so we have to move on, because we have to finish by a certain time. And that’s why I was saying that it would have been more useful to have shorter additional sessions on topics, because I think, you know, the instructors did feel like they had to finish by a certain time. And they did skip over a few things. (End User 13)

An effective training session was also characterised by equal participation of facilitators to moderate and mediate the discussion. Participants felt they were better able to engage with the session when technical difficulties arose because one facilitator was available for technical support while the other facilitator was presenting.

#### Adopt a peer-to-peer training approach

Although a collaborative curriculum development approach was used to design the training content, participants still felt that the content did not reflect their unique workflows and how they would interact with the system for their daily activities. However, completing the training did provide an effective overview of the foundational concepts required to use the HIS and was reflected in participants’ confidence in using the system. Learners were receptive to facilitators that provided role-specific examples to help with communicating key features of the platform even if it was out of the facilitator’s scope of prescribed scenarios:

They were comfortable in leading the classes. . . spoke clearly, they explained things, as well as they could they understood the current state in the department. So, they tried to make that link. Obviously, like everybody else, they are not privy to new workflows that will happen, because a lot of that is still work in progress. But to the best of their abilities, they tried to link the functionality to the workflow that’s currently happening in the department. . . (End User 5)

Therefore, to ensure that new workflows were understood, communicating examples from a peer was well received.

### Theme 2: Integrating practical learning activities that contribute to knowledge retention

#### Promote hands-on experiences to enhance learning opportunities

Learners appreciated how the sandbox environment supported the translation of foundational concepts to increase familiarity with the system and visualisation of workflow processes through completing scenarios:

Yeah, I think it was very hands-on, you know, it’s like, you have the thing in front of you. . . And, and the exercises that you went through, it really just gave you a handle on and how to navigate really, which I think basically is what you need. Because I mean, it’s, it’s to me, it’s user friendly. . . And you can play around still to a certain extent without, say, breaching, patient information, like just the tabs on top to see what’s there and to see if this is the information I need. But it’s fairly straightforward. I mean, now, but then yeah, it was still good to be able to navigate within the system while you’re being trained. (End User 18)

However, the time associated with exploring the sandbox was limited within the training session and learners reported that their work schedule restricted further experimentation. Learners expressed the need for more interactive learning opportunities during the session to address the lack of time post-training to explore the system:

We weren’t given much time to even play around in the system. Because you’re asking us to do training during our workday. And our workday doesn’t stop and wait for us. . . So, we didn’t have time to play around the system or even want to learn it that way. There was no engagement other than training. (Educational Champion 12)

To ensure that staff had adequate time to explore the system, participants stressed the importance of integrating time within the training session to focus on practical learning components.

#### Develop reinforcement learning approaches

Additionally, a few participants mentioned the scenarios were beneficial to reiterate key features within the system. Stronger knowledge retention was experienced by learners when facilitators used a repeat-back method for scenarios or summary documents:

And so, you know, we just actually would watch certain components, and she would go through all the steps. And then she would back up and say, Okay, so let’s go from step one, and then we would try it. So, I again, that just stuck in my head. (End User 13)

Another mechanism of reinforcement learning that took place included repeating training. Specifically, educational champions acknowledged that repeating training for the second time increased their confidence in the content and their ability to complete tasks using the HIS interface. Learners felt confident in completing the assessment of training when knowledge reinforcement and interactive activities were integrated throughout the training.

### Theme 3: Ensuring training resources are accessible and consistent for an optimal training experience

#### Deliver consistent training format and communication

Participants felt that email communications before the session provided a good mechanism to ensure the preparedness of training. Similarly, post-session emails were well-received because they contained answers to questions that were asked during the session. However, participants emphasised that inconsistencies in email communications impacted on how they engaged with the class and the resources they had access to

And so, I found that I have to say, number one, I love the fact that we get an email before with this is what you’re gonna be expecting. Here’s your course companion, here’s everything you need to know for the class. So that’s been wonderful. I did find it a bit inconsistent. Like there’s some that I’ve gotten like 2 days before the class, there’s some I’ve gotten the week before. So, it’s kind of like why am I getting this the week before and this, I’m getting only 2 days before. So, I did find the with some inconsistency there, of when we’re getting our documents and our information for the class. (Educational Champion 11)

Participants appreciated that the synchronous training session structure reflected the flow of the course companion, which helped them navigate through training. The following participant reflected on how the course companion was also a useful reference resource that helped learners engage and use the system post-training:

I actually liked the fact that they were virtual. I’ve attended a lot of training sessions that were in person, even here at [the organisation]. When I first started working here, I had to attend in training sessions for EPR, and PHS [previous clinical system]. So, I like the virtual format. I guess, also, the documents that we received in advance, they were pretty well organised. The teaching materials were very clear. And frequently, you could, you know, sort of supplement what you were hearing in sessions by reading outside of the session. And I’ve actually kept all my training materials so that I can create a sort of manual. (End User 12)

Participants felt that the training programme provided an adequate number of resources to supplement the content in the virtual setting while also adapting to their diverse learning needs.

#### Provide accessible technology resources and learning space

Several participants noted that during the first round of training sessions, learners required two monitors in order to actively engage in training. One to view the demonstration of the instructor and the other monitor to simultaneously mirror the activity in the training environment. This was to ensure a hands-on approach to training. Participants commented that access to computer labs onsite was effective in ensuring that learners had the technical resources necessary to engage in training seamlessly and efficiently with two monitors. Similarly, due to the variation in work schedules and shifts, onsite resources to complete training were beneficial. However, some learners who had access to technology at home expressed the benefits of being in a comfortable and familiar environment when undergoing long training sessions. Additionally, due to the virtual and hybrid components of some staff’s work schedules, participants reported they were more inclined to participate in their homes rather than onsite. Some participants mentioned technical support both within the session and from the support desk improved the learning experience:

Their responsiveness to your needs, I really, really appreciate that. They did a very good job in getting me started, which I thought. . . this is going to be a wasted day. And it wasn’t right. It turned out not to be. And it’s simply because they took me out, took me aside and say, Okay, what’s the problem? Let me help you. And they did help me. Right. And I, just when I needed for them, and the other nurse, I think there a couple of other nurses needed for them to slow down. They did and repeat whatever needed to be repeated they did they were very responsive. (End User 10)

Learners felt that the additional support to familiarise themselves with the video conferencing platform and upscale their digital skill level better prepared them to engage in virtual training.

## Discussion

By examining the opportunities and challenges through the peer-to-peer education and training strategy in this study, three themes were identified that contributed to the effective facilitation of HIS training in a virtual environment: (1) optimise the training experience by providing accessible and consistent training resources; (2) adapt to diverse learning needs by cultivating an inclusive learning environment; and (3) promote knowledge retention through practical learning activities. As healthcare organisations and educators adapt to this novel form of training, it is important to adopt a proactive approach to educate and prepare healthcare staff for the clinical transformation. However, very little literature is available on the effective practices and strategies for facilitating HIS training through a virtual delivery modality. The findings from a recent qualitative study by Jeyakumar et al. (2021), which assessed perceptions of virtual training for a HIS implementation among learners and educators, served as a foundation to further explore and identify key success factors that contribute to effective knowledge transfer of HIS competencies through virtual training. The findings were situated based on the Accelerating the HIS Learning Cycle framework for HIS education design and development and the Kirkpatrick–Barr framework.

### Integrate adult learning principles

The findings from this evaluation emphasise the importance and need to foster dynamic facilitation techniques to cultivate an inclusive culture and adapt to the diverse learning needs of staff. This recommendation aligns with the first phase of the Accelerating the HIS Learning Cycle where it is important to assess the individual, team, and organisational capabilities to deliver training. It is especially important in a large-scale HIS implementation where different experiences, skillsets and identities are brought together into one virtual classroom. Integrating adult learning principles into the training curriculum is an avenue to adapt to the emerging learning needs of staff and create an engaging learning environment.

Adult education literature suggests that adult learners receive information best when it is seen as relevant to their work, builds on existing knowledge and requires active engagement (Collins, 2004). The education strategy used in this study strove to create content relevant to diverse workflows by leveraging a peer-to-peer training approach, co-creating curriculum with staff, and ensuring facilitators integrated hands-on activities to transcend the imposed social barriers of a virtual learning space. The study effectively demonstrated how integrating adult learning principles achieved both Levels 1 and 2 education outcomes to improve staff satisfaction with the training and influenced a positive perception of using the HIS in their practice. Adult learning principles were intentionally woven into our HIS training through facilitating peer-to-peer mentorship and curriculum creation to promote real-world situations during training. A study by Lawrence et al. (2020) supported this finding as it yielded an increase in satisfaction among staff when their paediatric hospital’s HIS curriculum was redesigned to incorporate adult learning principles and was delivered by peers. Similar to our study, the Lawrence et al. (2020) study highlighted the importance of cultivating a stress-free and accessible learning environment to troubleshoot areas of difficulty and constructively focus on areas relevant to staff’s unique workflows.

### Practical learning activities and PBL approach

The current study further highlights the need for integrating practical learning activities that contribute to knowledge retention, including access to the sandbox environment, providing real-life scenarios, and incorporating knowledge assessments. The HIS learning cycle addresses that practice, and PBL is an important component of HIS education. Participants from this study stressed the need for hands-on practice as it enabled them to become familiar with the system and to understand the clinical workflows. Additional studies have demonstrated that knowledge retention is supported by reiterating key functionalities within the system (Bredfeldt et al., 2013; Greenhalgh, 2001; Kersey and Robinson, 2018). Findings from our study further build on the importance of reinforcement, as educational champions emphasised that repeating the training for the second time allowed for knowledge consolidation and increased their confidence in using the system. These findings were further corroborated by Kersey and Robinson (2018). These authors asserted that providing HIS education in a hands-on workshop setting resulted in improved performance and well-being (Kersey and Robinson, 2018). Optimising the delivery of education has a significant impact on care delivery by improving their critical thinking skills and decreasing the time spent in the system (Kersey and Robinson, 2018). Similarly, Bredfeldt et al. (2013) reported that hands-on exercises were a contributing factor to the training’s success.

A PBL approach is a way to activate practical learning activities as it cultivates an active independent learning attitude among learners and enables them to internalise fundamental concepts learned to promote critical thinking skills (Takahashi, 2008). Further, it reinforces knowledge retention and facilitates knowledge transfer to clinical tasks (Mansur et al., 2014). A PBL approach was used in the organisation’s virtual HIS training to facilitate level three education outcomes of the Kirkpatrick–Barr model in order to encourage learners to change their behaviour outside of the learning environment and effectively adopt HIS workflows. In the context of the HIS implementation, this approach included working in the sandbox environment to complete case studies and tasks in order to change the behaviour of learners, adopt the system, and the associated new workflows. Key characteristics of PBL include learner-centeredness, dialogic and collaborative learning (Mansur et al., 2014). In comparison to task-based approaches, PBL requires a shift to a learner-centred and inquiry-based environment (Mansur et al., 2014). Takahashi (2008) argued that this approach encourages lifelong learning skills, which will be beneficial in navigating clinical workflows and iterative updates to the system. Vuk et al. (2015) affirmed that interactive activities such as simulation training and exploring the HIS interface better position staff to translate lessons learned directly into practice and increase the level of self-confidence, and preparedness to use the HIS. The act of simulation improves the staff’s self-efficacy and the ability to use the HIS after training (Vuk et al., 2015).

### Accessibility and consistency of training resources

The study emphasised the need to ensure training resources are accessible and consistent to address the first phase of the HIS Learning Cycle where education should be adapted to and for the staff’s learning needs. For an optimal learning experience, organisations may need to consider how to create accessible learning resources by providing them in multiple formats, facilitating technical support, and addressing barriers to accessing technology and learning space. Learners in our study said that the course companion effectively reflected the flow of the synchronous training structure, serving as a reference tool post-training. This approach worked towards achieving level 1 outcomes of the Kirkpatrick–Barr framework, which focused on the satisfaction of learners with the training strategy. Smailes et al. (2019) identified barriers that could hinder satisfaction in a virtual training environment included not having enough time to complete the content, a preference for content being delivered as instructor-led training, and too slow of a pace if experience level with using the electronic health record varied. Adapting to diverse learning needs included providing access to computer labs on site, which was deemed effective in ensuring learners had technical resources and available space to engage in training efficiently with two monitors. This further supports the creation of equitable and inclusive learning spaces, particularly in a virtual context. One of the key success factors for a training program is to ensure an accessible and comfortable training location (Nicklaus et al., 2015). The accessibility of training also focused on increased responsiveness and timely support from the IT help desk, allowing experts to help learners navigate through technical issues during training (Box 1).

**Box 1.** Kirkpatrick–Barr training evaluation framework.

Key themes and subthemes	Kirkpatrick’s training evaluation model		
	L1 reaction	L2 learning	L3 behaviour
Theme 1: Foster dynamic facilitation techniques to cultivate an inclusive culture and adapt to diverse learning needs	√	√	
Theme 2: Integrate practical learning activities that contribute to knowledge retention	√	√	√
Theme 3: Ensure training resources are accessible and consistent for an optimal training experience	√	√	

### Future directions

Informed by the learning cycle framework by Jeyakumar et al. (2021), this study was beneficial in assessing the effectiveness of virtual training for a HIS implementation. However, it is important to continually address ongoing learning needs for future iterations of HIS education programs to better understand how providers can successfully adopt technology into practice. In order to do so, future efforts should focus on expanding the third level of the Kirkpatrick–Barr framework to explore the long-term retention of training material and examine practice changes in training delivery. Additionally, the fourth level of Kirkpatrick–Barr framework should be assessed by ensuring adequate support available to stakeholders engaging in virtual training while also investigating the impact of HIS implementation in a clinical environment with staff and patients. As there are still key practice and knowledge gaps to support learners in the design, development and use of training tools, ongoing research should address effective education approaches for the virtual training modality of HIS implementation (Jeyakumar et al., 2021).

### Limitations

Findings of this study must be examined within the context of the following limitations. The study was limited due to the alignment with the COVID-19 waves in Canada. This could have impacted participation levels in both the surveys and interviews due to the rise in burnout and increased demand for clinical staff to be available to work. To mitigate this, we offered multiple rounds of interviews to adhere to the staff’s schedules and offered a token of appreciation to encourage participation. Although the study used purposive sampling to be as inclusive as possible, participants were all still localised in a Canadian context. However, the study design interviewed and surveyed staff at three hospital sites to ensure full coverage of perspectives. Another limitation was that participants might have been more likely to engage in research activities and interviews if they had constructive feedback rather than those who were satisfied with the HIS training program. Finally, recognising that researchers’ positions and perspectives inevitably influence access to findings, we asserted research rigor and reflexivity through triangulation of the data from multiple perspectives.

## Conclusion

Our study findings, guided by the HIS learning cycle, validated the virtual learning approach as a feasible method of delivery for HIS education and implementation. This evaluation has the potential to inform key success factors to support learner needs for virtual environments for learning clinical systems. Education is an important driver for innovation in technology including the success of digital transformations across an organisation. Effectively developing a virtual learning environment that values continuous quality improvement to learners’ needs will contribute to the long-term success of future iterations of HIS adoption. This evaluation can further inform the development of HIS virtual learning programs, which can equip healthcare providers in performing their tasks; successfully adopting the most effective education strategies with the skills required to work in a clinical environment.

## Notes

1. ‘Sandboxing’ is cybersecurity term for virtual space that mimics a user operating environment, while remaining isolated from the main system (Almunawar and Anshari, 2012).
